# MicroRNA expression profiling and functional analysis of CDH3 during oogenesis in the Chinese alligator (*Alligator sinensis*)

**DOI:** 10.1093/cz/zoaf058

**Published:** 2025-08-21

**Authors:** Changcheng Li, Yuan Zhang, Yue Wen, Chong Wang, Pengfei Li, Yunlu Xu, Yuqian Zhang, Yongkang Zhou, Xiaobing Wu, Haitao Nie

**Affiliations:** The Anhui Provincial Key Laboratory of Biodiversity Conservation and Ecological Security in the Yangtze River Basin,Collaborative Innovation Center of Recovery and Reconstruction of Degraded Ecosystem in Wanjiang Basin Co-Founded by Anhui Province and Ministry of Education, Anhui Normal University, Wuhu, Anhui Province 241000, People's Republic of China; The Anhui Provincial Key Laboratory of Biodiversity Conservation and Ecological Security in the Yangtze River Basin,Collaborative Innovation Center of Recovery and Reconstruction of Degraded Ecosystem in Wanjiang Basin Co-Founded by Anhui Province and Ministry of Education, Anhui Normal University, Wuhu, Anhui Province 241000, People's Republic of China; The Anhui Provincial Key Laboratory of Biodiversity Conservation and Ecological Security in the Yangtze River Basin,Collaborative Innovation Center of Recovery and Reconstruction of Degraded Ecosystem in Wanjiang Basin Co-Founded by Anhui Province and Ministry of Education, Anhui Normal University, Wuhu, Anhui Province 241000, People's Republic of China; The Anhui Provincial Key Laboratory of Biodiversity Conservation and Ecological Security in the Yangtze River Basin,Collaborative Innovation Center of Recovery and Reconstruction of Degraded Ecosystem in Wanjiang Basin Co-Founded by Anhui Province and Ministry of Education, Anhui Normal University, Wuhu, Anhui Province 241000, People's Republic of China; The Anhui Provincial Key Laboratory of Biodiversity Conservation and Ecological Security in the Yangtze River Basin,Collaborative Innovation Center of Recovery and Reconstruction of Degraded Ecosystem in Wanjiang Basin Co-Founded by Anhui Province and Ministry of Education, Anhui Normal University, Wuhu, Anhui Province 241000, People's Republic of China; The Anhui Provincial Key Laboratory of Biodiversity Conservation and Ecological Security in the Yangtze River Basin,Collaborative Innovation Center of Recovery and Reconstruction of Degraded Ecosystem in Wanjiang Basin Co-Founded by Anhui Province and Ministry of Education, Anhui Normal University, Wuhu, Anhui Province 241000, People's Republic of China; The Anhui Provincial Key Laboratory of Biodiversity Conservation and Ecological Security in the Yangtze River Basin,Collaborative Innovation Center of Recovery and Reconstruction of Degraded Ecosystem in Wanjiang Basin Co-Founded by Anhui Province and Ministry of Education, Anhui Normal University, Wuhu, Anhui Province 241000, People's Republic of China; Alligator Reproductive Research Center of Anhui Province, Xuanzhou 242000, People's Republic of China; The Anhui Provincial Key Laboratory of Biodiversity Conservation and Ecological Security in the Yangtze River Basin,Collaborative Innovation Center of Recovery and Reconstruction of Degraded Ecosystem in Wanjiang Basin Co-Founded by Anhui Province and Ministry of Education, Anhui Normal University, Wuhu, Anhui Province 241000, People's Republic of China; The Anhui Provincial Key Laboratory of Biodiversity Conservation and Ecological Security in the Yangtze River Basin,Collaborative Innovation Center of Recovery and Reconstruction of Degraded Ecosystem in Wanjiang Basin Co-Founded by Anhui Province and Ministry of Education, Anhui Normal University, Wuhu, Anhui Province 241000, People's Republic of China

**Keywords:** CDH3, Chinese alligator, gonads, miRNAs

## Abstract

MicroRNAs (miRNAs), noncoding RNAs that regulate the expression of target mRNAs, have gained attention. Nevertheless, the biological mechanism underlying oogenesis in crocodiles remains unclear. In this study, RNA sequencing was utilized to analyses miRNA expression at 1-, 15-, and 90 days post-hatching (dph) in *Alligator sinensis*. We identified 92 differentially expressed known miRNAs. Among these genes, 17, 1, and 5 had specific expression patterns at 1, 15, and 90-dph, respectively. GO and KEGG analyses of the predicted miRNA targets revealed enrichment in cell adhesion molecule (CAM) pathways. The expression of CDH3 was significantly higher than that of other family members and was high during the embryonic stage, which coincided with the commencement of mammalian oogenesis. Our prediction indicated the presence of 3 Ca^2+^-binding sites, 2 cadherin domains, and a cadherin cytoplasmic region in the CDH3 amino acid sequence. This finding suggests a similar cell adhesion function to that of mammalian CAM family genes. IHC analysis revealed minimal CDH3 expression in the germ cell nests at 1-dph. Elevated CDH3 expression was observed in primordial follicles formed via Nest breakdown at 15-dph. Notably, CDH3 expression decreased significantly at 90-dph, but positive signals remained in thecal epithelia of the medullary cavities. The results of lentivirus experiments revealed that CDH3 downregulation suppressed the expression of oogenesis genes (FSHR, CYP19A1, BMP15, and BCL2). Our research highlights the function of miRNAs in crocodilian oogenesis, and CDH3 is proposed to be crucial for reproductive developmental mechanisms.

## Graphical Abstract

**Graphical Abstract:**
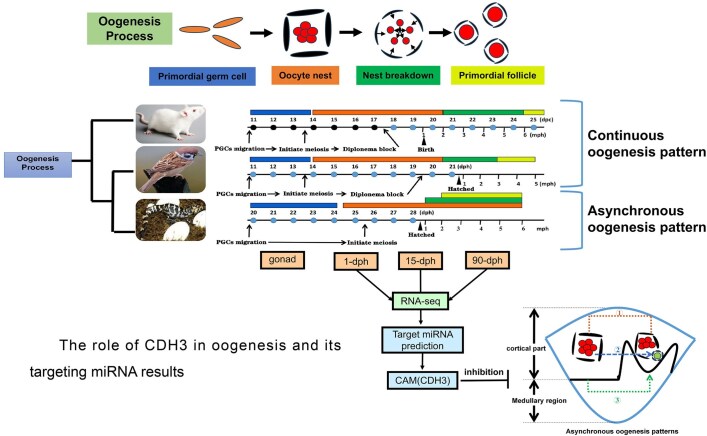

The reproductive lifespan of female mammals is determined at birth. The formation of primordial follicles, each consisting of an oocyte surrounded by a layer of somatic cells, is a necessary condition for fertility (Pepling 2012). In mice, primordial germ cells originate from the primitive ectoderm layer and, at embryonic day 9.5, embark on their migration toward the genital ridges via the dorsal mesentery (Saitou and Yamaji 2012). Interestingly, in Caiman crocodilus, the entrance of oogonia into meiotic prophase has been consistently observed throughout the reproductive lifespan of the reptile (Calderón et al. 2004). Furthermore, studies on the American alligator (*Alligator Mississippians*) have shown that the morphology of yolk platelets during the mid- and late stages of oogenesis resembles that seen in birds (Uribe and Guillette 2000). Additionally, recent research has confirmed that the initiation of the differentiation transition in oocytes of the Chinese alligator (*Alligator sinensis*) occurs on the 15th day post-hatching (Nie et al. 2023). However, the cellular and molecular mechanisms of the oogenesis process in crocodile species are less known. MicroRNAs (miRNAs) are small RNAs (sRNAs) that regulate gene expression by targeting mRNAs, and they participate in oogenesis (Tesfaye et al. 2018; Salilew-Wondim et al. 2020). Investigations of how miRNA expression affects oogenesis have been conducted primarily in mammals (Zhang et al. 2013; Gilchrist et al. 2016; Wang et al. 2018; Kataruka et al. 2020; Liu et al. 2021b), fish (Gay et al. 2018; Alvi et al. 2021; Ma et al. 2021), and birds (Ocłoń and Hrabia 2021; Li et al. 2023; Yu et al. 2023).

Cadherin is a transmembrane protein belonging to a superfamily of calcium-dependent membrane glycoproteins involved in cell adhesion (Chan 2006). Cadherins play crucial roles in the establishment and progression of oocytes and follicles. CDH1 (E-cadherin) contributes significantly to the assembly of nests (Ikami et al. 2023) and regulates the timing of oocyte migration from nests (Wang and Roy 2010). It also maintains the primordial follicle pool (Yan et al. 2019). The pivotal expression of CDH2 (N-cadherin) is essential for pregranulosa cell adhesion during primordial follicle formation (Wang and Roy 2010). U-cadherin can form a junction between oocytes and follicular cells in the African clawed frog (Müller et al. 1992).

The Chinese alligator (*Alligator sinensis*), an endangered species endemic to China, is considered a “living fossil” owing to its unique evolutionary history (Pan et al. 2019). Studies on the miRNAs of the Chinese alligators have focused mainly on the processes of temperature-dependent sex determination and differentiation (Lin et al. 2018), the importance of hibernation to ovarian development (Lin et al. 2020a, 2020b; Zhang et al. 2021a), and the epigenetic mechanisms of sex maintenance(Hu et al. 2022). We constructed a miRNA expression profile of the ovaries of the juvenile Chinese alligator, aiming to establish an mRNA-miRNA regulatory network and identify key miRNAs involved in oogenesis. These data improve our understanding of the mechanisms involved in oogenesis in crocodilians, specifically Chinese alligators, and contribute to their conservation.

## Materials and methods

The animal protocols employed in this study were in accordance with the Measures for the Administration of the Permit for Experimental Animals, as outlined by the Ministry of Science and Technology of the People's Republic of China (2nd ed. No. 593, 2001). Additionally, the procedures for humane animal care and handling were approved by the Guide for the Care and Use of Laboratory Animals, as prepared by the Ethics Committee of Anhui Normal University (Project Identification Code: No. AHNU-ET2021008; Approval Date: 6 March 2021).

### Animal tissue and sample collection

Nine Chinese alligator eggs were obtained from Wuhu Dajiang Farm, (Wuhu, Anhui, China; longitude 118.41°, latitude 31.29°). All eggs were carefully transferred to the Chinese Alligator Research Center in Anhui Province and hatched in wet moss at 29 °C, 90% humidity. The entire incubation period lasted 70 days, A total of 9 crocodile eggs successfully hatched. After identifying the individuals based on the characteristics of their head scales, one of the first hatched eggs (referred to as the 1-day group, 1-dph) was randomly selected on the first day after hatching and euthanized with excessive anesthesia. The remaining crocodiles were fed artificially after hatching and were euthanized with anesthesia on the 15th day (15-dph) and 90th day (90-dph) after hatching, respectively. All the individuals were euthanized via decapitation following deep anesthesia with an intraperitoneal injection of sodium pentobarbital (50–100 mg/kg, Propbs, Beijing, China). Furthermore, the gonadal tissues were divided into left and right halves. The remaining gonadal tissues were placed in 15-mL centrifuge tubes prefilled with 4% paraformaldehyde solution and kept at 4 °C in refrigerator overnight. The gonadal tissues were extracted, placed into liquid nitrogen for quick freezing, and transferred to −80 °C until use.

### RNA extraction, sRNA library preparation, and sequencing

Total RNA was extracted from gonads via Trizol RNA Isolation Kit (Invitrogen, Waltham, MA) for the construction of sRNA libraries at 1-dph, 15-dph, and 90-dph. RNA integrity was assessed via the RNA Nano 6000 Assay Kit of the Agilent Bioanalyzer 2,100 system (Agilent, Santa Clara, CA) to evaluate RNA quality, and a library was constructed for the qualified total RNA (RIN ≥8.0). For each sample, 3 µg of RNA was used to construct sRNA libraries via the NEBNext® Multiplex Small RNA Library Prep Set for Illumina® (New England Biolabs, Ipswich, MA), according to the manufacturer's instructions. The library preparations were subsequently sequenced on an Illumina HiSeq 2,500/2,000 platform and 50 base pair single-end reads were generated.

### Sequencing data analysis

After conversion of the sequencing fragments into the original sequence data (reads), reads containing adaptor sequences, “N” bases, and those with low quality (e.g., those in which the proportion of bases with a quality value of ≤5 exceeded 50%, or that had an error rate > 1%) were removed. Filter out polyA-rich reads, and tags with low abundance (<2 counts). rRNA, rRNA, scRNA, snoRNA, snRNA, and tRNA contaminants were identified and removed using GenBank and blastall 2.2.25 (blastn, identity ≥ 97%). Clean reads were aligned to the Chinese alligator reference genome (RefSeq: GCF_000455745.1) via Bowtie 2.2.3 (mismatch = 1) (Langmead and Salzberg 2012). Reads mapped to the reference sequence were compared with the specified range sequence in the miRBase to identify known miRNAs. New miRNAs were analyzed by means of miREvo (Wen et al. 2012) and mirdeep2 (score = 4) (Friedländer et al. 2012). The TMM (number of transcripts mapped to miRNA per million transcripts) was used to standardize the read count data, and then DEGseq was used for differential analysis (Wang et al. 2010). The criteria for screening differential miRNAs were as follows: q value < 0.01 and |log2(fold change) | > 1. The relationships between miRNAs and target genes were evaluated via miRanda (parameters: -sc 140-en-10-scale 4-strict) (Enright et al. 2003) and RNAhybrid (parameters: -e-10-p 0.05-m 50000) (Rehmsmeier et al. 2004). KEGG pathway and GO enrichment analyses were performed on the target genes of the differentially expressed miRNAs in each group via KOBAS (Mao et al. 2005) and GOseq (Young et al. 2010) (*P* ≤ 0.05).

### Transfection assay

Gonadal tissues from three 15-dph Chinese alligators (*n* = 3) were cultured in accordance with previously described methods (Shen et al. 2007). Cultured gonadal tissues were transfected with either shRNA (GenePharma Co., Ltd. Shanghai) using metafectene (Biontex, TO20-1.0, Munich, Germany) following the supplier's instructions. At the 48th hour, half of the medium was replaced with complete medium. The lentivirus used to infect the gonadal tissues contained a CDH3-shRNA sequence (5′-GTGACTTTGTCGTGTGCAATC-3′) (GenePharma Co., Ltd. Shanghai). All data were obtained from paired gonadal tissues (for 2 gonadal tissues of a Chinese alligator, one was transfected with target shRNAs vector and the other was transfected with nontargeting control).

### Immunofluorescence

After paraformaldehyde fixation and dehydration in a series of graded ethanol solutions, all the paraffin-embedded sections (6 μm thick) were deparaffinized in xylene and further exposed to citrate antigen retrieval solution in a microwave at 100 °C for 15 min to activate the tissue surface antigens. After treatment with immunostaining blocking buffer for 1 h, the sections were incubated overnight at 4 °C with primary antibodies against CDH1 (Bioss, bs-10009R), CDH2 (Bioss, bs-20622R), and CDH3 (Bioss, bs-1159R) at a dilution of 1:1,000. The next day, the slides were washed twice in PBS and incubated with secondary antibody at a dilution of 1:100 at 37 °C for 1 h. After the secondary antibody was thoroughly washed off the specimen with PBS, the sections were stained and then counterstained with hematoxylin. Images were captured via electron microscopy (Leica Microsystems, Wetzlar, Germany).

### Western blot

Tissue samples were lysed in RIPA lysis buffer (Sangon, C510006) to collect total protein. The proteins were separated by SDS-PAGE and transferred to a PVDF membrane (Sangon, F619534). The membrane was blocked for 1 h at room temperature in TBST containing 5% BSA. The samples were then incubated overnight at 4 °C in TBST containing 5% BSA with anti-CDH3 monoclonal rabbit antibody (Bioss, bs-1159R, diluted 1:1,000) and anti-GAPDH monoclonal rabbit antibody (Bioss, bs-10900R, diluted 1:10,000) separately. The membrane was subsequently washed in TBST and incubated with a secondary antibody conjugated with horseradish peroxidase (Beyotime, A0208, diluted 1:500) for 1 h at room temperature. After washing with TBST, the bands were detected with the a SuperSignal chemiluminescence kit (Beyotime, P0018FS). The intensity of each band was quantified with GAPDH as the reference via image analysis software. Three biological replicates were conducted.

### Cloning of the CDH3 cDNA coding region

The reverse transcriptase reaction was conducted according to the instructions provided with the PrimeScript 1st cDNA Synthesis Kit (Tiangen Biotech, China). The primers utilized are detailed in the Supplementary Material (Supplementary Table S1) and were designed on the utilized accession number XM_025209138.1, which was retrieved from the NCBI database. Total cDNA from the gonadal tissues of Chinese alligators was used as a template for PCR. All primers used in this study were designed via Primer Primer 50 and synthesized by Sangon Biotech (Shanghai). The PCR products were subsequently purified via a DNA gel extraction kit (Axygen, Hangzhou), and the purification products were subcloned and inserted into PMD-18 T vectors (TaKaRa, Dalian), followed by transformation into DH5α *Escherichia coli* cells (TransGen, Beijing). A total of 9 to 10 clones were identified as positive and selected at random for DNA sequencing (Shanghai, Sangon).

### Real-time quantitative PCR

Total RNA was extracted from the gonadal tissues of the 1-dph, 15-dph, and 90-dph samples and reverse-transcribed into cDNA using the PrimeScript RT Master Mix (Takara, Beijing, China) according to the manufacturer's instructions. The primers were designed via Primer Premier 5.0, the corresponding information including the GenBank accession numbers and primer sequences for the target and internal control genes, is listed in Supplementary Table S2. To confirm that the amplification efficiencies were close to 100%, melting curves were used to validate all the designed primers. All samples were analyzed in triplicate from the same RNA preparation and the mean values were calculated. Relative gene expression levels were calculated via the 2^−ΔΔCT^ method with ribosomal protein L8 (RPL8) used as the internal reference gene.

### Statistical analysis

All the statistical analyses were conducted with SPSS version 19.0 (Chicago, IL, USA). The significance level was set at a = 0.05 to identify statistically significant differences. A paired *t*-test was used to assess the concordance between the SDS-PAGE and qPCR results. The final data are presented as the mean ± SEM.

## Results

### Transcriptome sequencing data quality assessment

The quality of the raw sequencing data for each sample is statistically analyzed, typically using Q20 (base sequencing error rate of 1%) and Q30 (base sequencing error rate of 0.01%) to assess data quality. The distribution of GC content is examined to determine if there is any GC bias, which helps in assessing the randomness of the sequencing process. The results show that the Q20 ratio for all samples is over 99%, and the Q30 ratio is over 97%. The GC content ranges from 48.51% to 51.72%, with no significant GC bias observed. The sequencing error rate, calculated using a formula, is maintained at 0.01%. All data indicate that the quality of the sequencing data is high. The statistical results of the raw sequencing data quality for each sample are detailed in Supplementary Table S3 (Supplementary Table S3).

**Figure 1:**
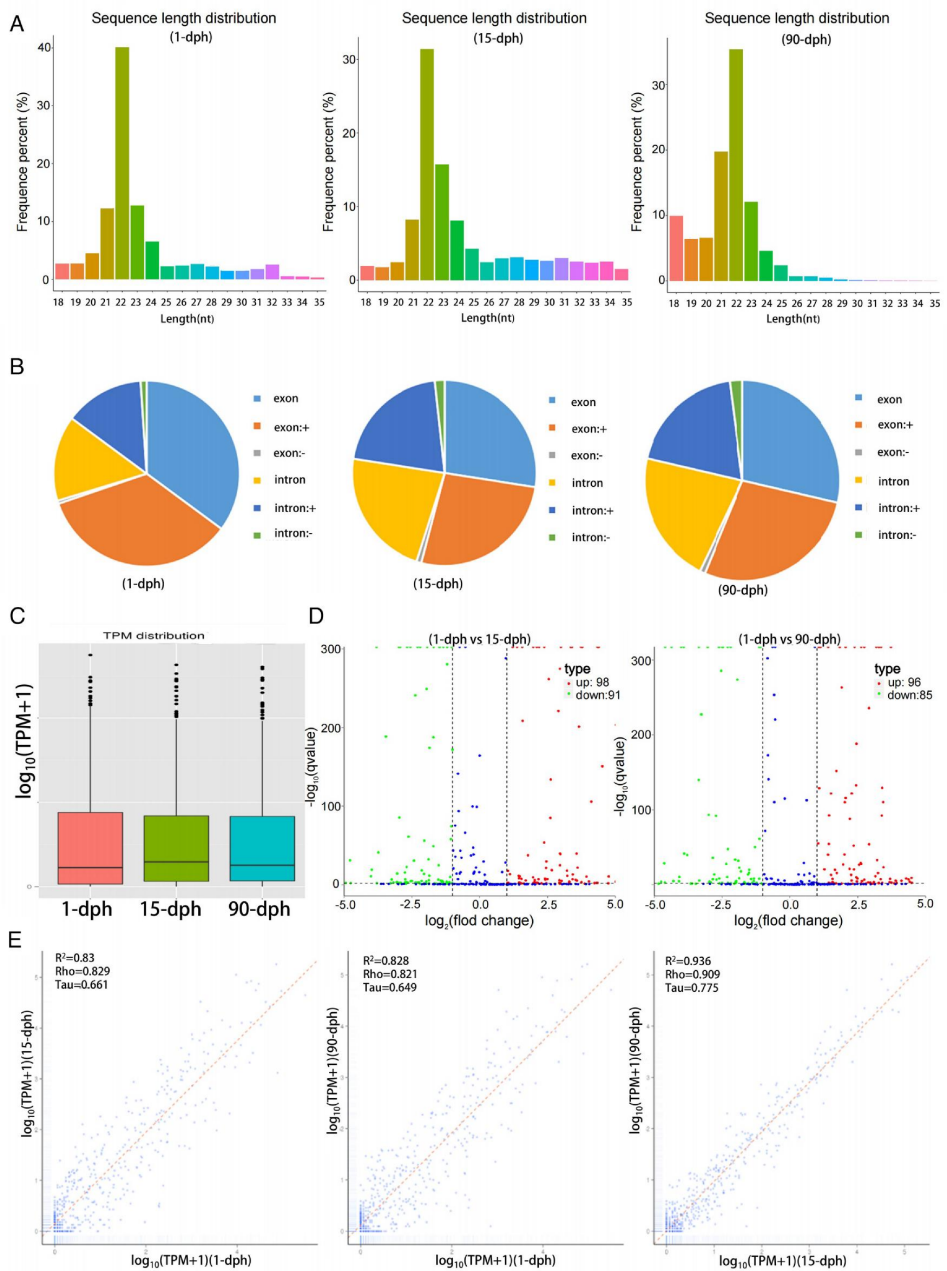
Analysis of miRNA differential expression during the 1-dph, 15-dph, and 90-dph periods: (A) Statistical analysis of sRNA species, abundance, and length distribution within a defined length range. (B) sRNA mapping onto gene exons and introns across different samples. (C) The TPM values of miRNAs at 1-dph, 15-dph, and 90-dph. (D) Differentially expressed miRNAs were identified in 1-dph vs. 15-dph, and 1-dph vs. 90-dph. (E) miRNA expression correlation between 1-dph vs. 15-dph, 1-dph vs. 90-dph, and 15-dph vs. 90-dph.

A comparison of the genome distribution of small RNAs revealed that the enrichment of sRNA in specific regions of some chromosomes suggests their involvement in specific patterns of gene expression regulation (Supplementary Figure S1 of the Supplementary Information). As shown in Fig. 1A, the majority of the sRNAs were 21–23 nt in length, with 22 nt being the most common size class (Fig. 1A). After screening, the sequences obtained by sequencing were found to be located mainly on the sense strand of the chromosome (Fig. 1B). We observed a greater abundance of repeat-associated reads for small RNAs, with sRNA species matching LTRs, LINEs, and DNA repeats and fewer significant differences in other categories (Supplementary Figure S2 of the Supplementary Information). Moreover, predictions of the novel miRNAs in the sample are provided in the Supplementary material (Supplementary Figure S3). The quality of our RNA-Seq data indicated that the samples were of high quality.

### miRNA differential expression analysis

**Figure 2:**
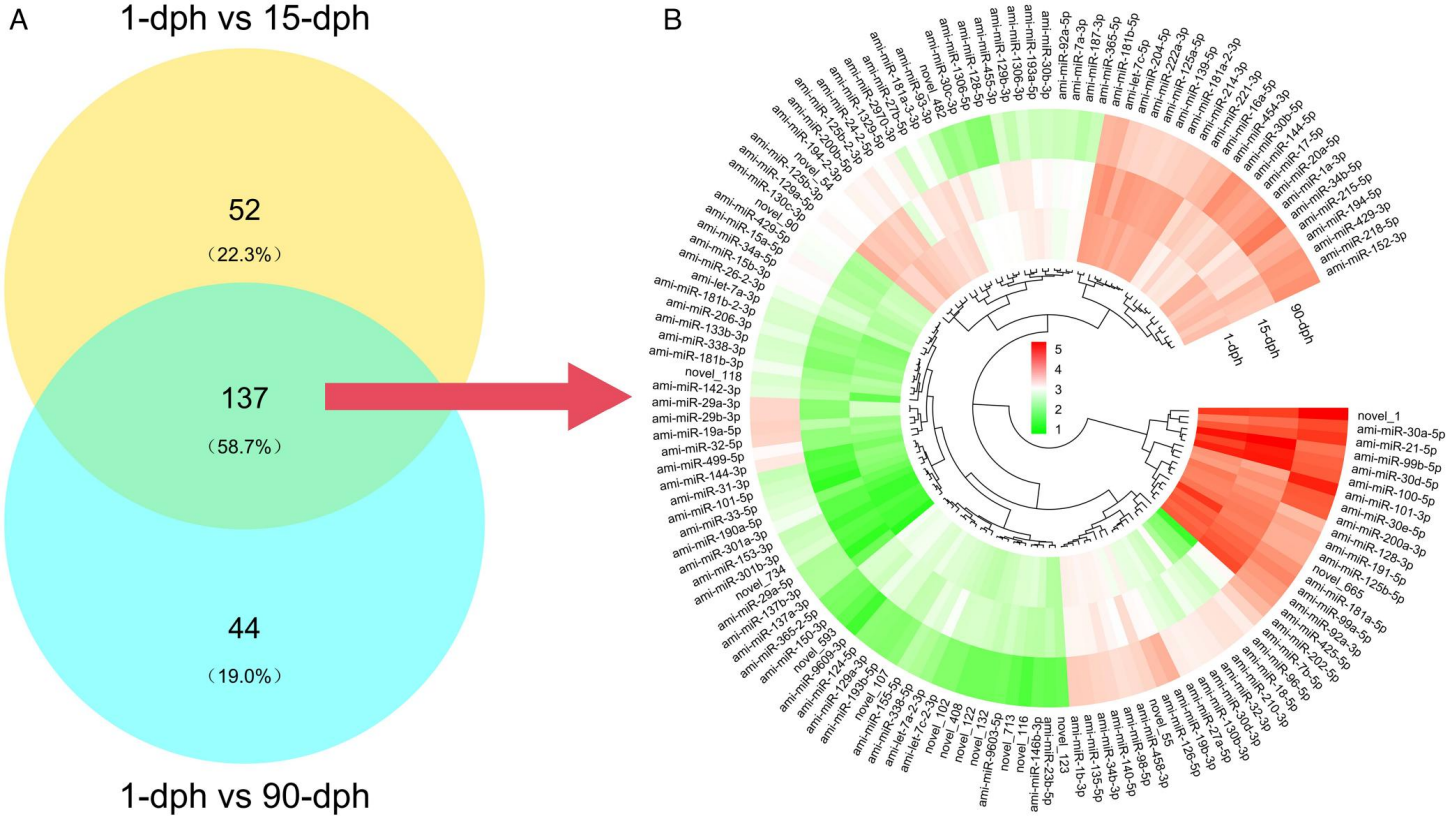
Identification of differentially expressed miRNAs: (A) Venn diagram of differentially expressed miRNAs between 1-dph vs. 15-dph and 1-dph vs. 90-dph.(B) Heatmap of differentially expressed miRNAs.

As shown in Fig. 1C, in our analysis, we compared the miRNA TPM density distributions across the 3 stages and observed that miRNA expression was higher at 15-dph. The Pearson correlation coefficient between 15-dph and 90-dph was 0.936, indicating high similarity (Fig. 1E). To identify differentially expressed miRNAs across the 3 stages, DESeq software was used to compare the changes in miRNA expression. Among the comparisons, in the 1-dph vs. 15-dph comparison, 91 miRNAs were downregulated and 98 were upregulated. Similarly, in the 1-dph vs. 90-dph comparison, 85 miRNAs were downregulated, and 96 were upregulated (*P* < 0.05; Fig. 1D). As shown in Fig. 2, 137 DEMs were expressed in 1-dph vs 15-dph and 1-dph vs 90-dph groups (Fig. 2A). Furthermore, the expression pattern of the DEMs in the groups was visualized using a cluster heatmap (Fig. 2B), revealing contrasting patterns of expression between treatments.

**Figure 3:**
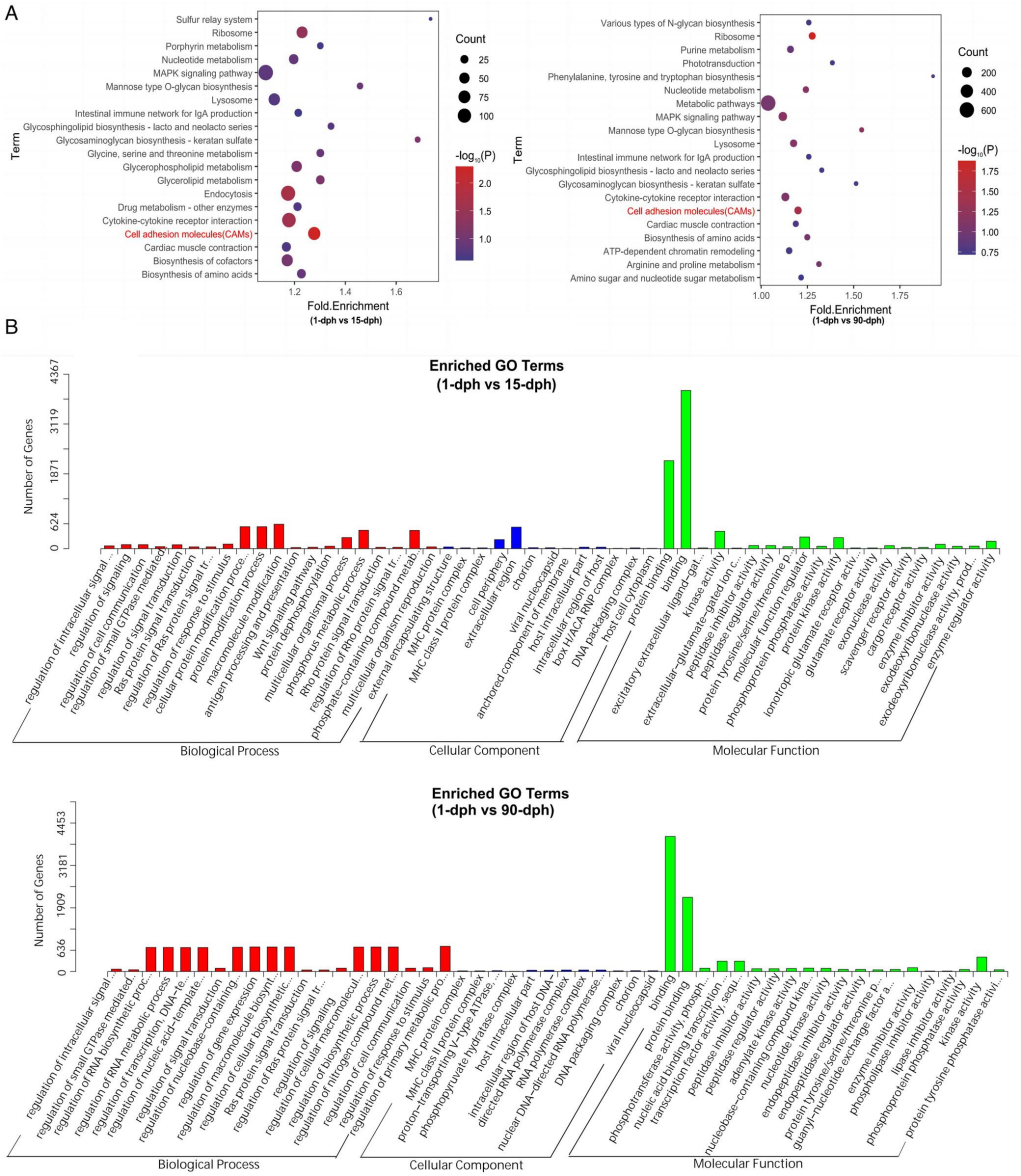
GO and KEGG pathway enrichment analysis of the predicted target genes. (A) KEGG pathway enrichment analysis of the predicted target genes. (B) GO annotation of the predicted target genes of differentially expressed miRNAs.

We predicted the target genes of the differentially expressed miRNAs to explore their potential functions. GO categories were identified (Fig. 3B). Biological processes, cellular processes, and molecular functions were enriched during the three stages. The number of genes related to “binding” molecular functions was the highest in both the 1-dph vs. 15-dph and the 1-dph vs. 90-dph comparisons. The differentially expressed miRNAs in the 1-dph vs. 90-dph comparison, in terms of their biological processes, were enriched in various metabolic processes, such as nitrogen compound metabolic process, cellular aromatic compound metabolic process, organic substance metabolic process, and primary metabolic process (Fig. 3B). KEGG is a signaling pathway database that includes a comprehensive pathway map, and 154 pathways were identified during the three stages (Fig. 3A). Interestingly, KEGG pathway analysis revealed that the cell adhesion molecule (CAM) pathway was enriched in both the 1-dph vs. 15-dph and 1-dph vs. 90-dph comparisons (*P* < 0.05; Fig. 3A). Other pathways, such as the MAPK signaling pathway, were also identified as closely related to the metabolic pathways that may contribute to the development of the three stages.

### miRNA family analysis

Family analysis of the detected known and novel miRNAs was performed to explore the presence of their respective miRNA families in other species. Some of the results are presented in Supplementary Table S3. Most miRNA families associated with ovun development, such as let-7, miR-134, miR-145, miR-182, and miR-383, are expressed in reptiles and birds such as Alligator mississippiensis, Anolis carolinensis, Chrysemys picta, Columba livia, Gallus gallus, Python bivittatus, and Xenopus tropicalis, suggesting strong conservation (Supplementary Table S4).

### Expression patterns of CDH3 in the gonads across different developmental stages

**Figure 4:**
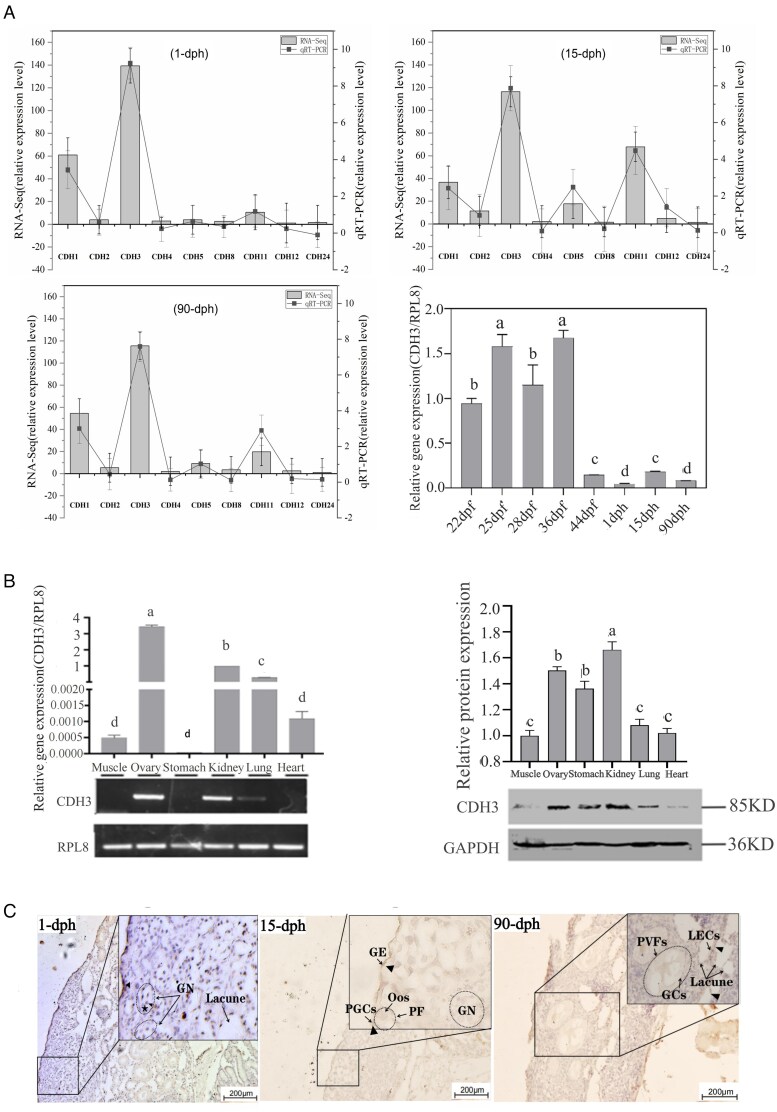
Temporal and spatial mRNA expression patterns o CDH3 within gonads: (A) RT-qPCR-based verification of the expression of cadherin mRNA and temporal expression analyses of CDH3. (B) The tissue expression profile of CDH3 at 15-dph. (C) Immunohistochemical localization of CDH3 in the gonad of the Chinese alligator. ★: oogonia, ▴: Positive expression sites, dpf (days post-fertilization), dph (days post-hatch), (Mean ± SEM; Error bars are indicative of the standard error). GN, germ nest; GE, germinal epithelium; Oos, oocytes; PGCs, pre-granulosa cells; PF, primordial follicle; PVFs, pre-vitellogenic follicles; LECs, lacunar epithelial cells; GCs, granulosa cells.

During the verification of the CAM signaling pathway enriched with gene expression data from the CDH family (Fig. 4A), we observed that CDH3 had a significantly higher level of expression than other family members did (*P* < 0.05; Fig. 4A), significantly elevated expression in the gonadal complex (ovaries and kidneys combined) at 15-dph (*P* < 0.05; Fig. 4B), and a high expression phase during the embryonic stage (*P* < 0.05; Fig. 4A). This finding is consistent with the biological phenomenon of mammalian oogenesis starting at this stage (Tingen et al. 2009). Therefore, we conducted in-depth research and analysis on the CDH3 gene in the CAM pathway.

We found that the minimal expression of CDH3 at 1-dph (*P* < 0.05; Fig. 4A), which was predominantly localized to nests, suggests its role in nest stability, where the low expression level may indicate nest dissociation (Fig. 4C, left panel). At 15-dph, the elevated expression of CDH3, was predominantly concentrated in primordial follicles after nest breakdown (Fig. 4C, middle panel), indicating its involvement in mediating oocyte–granulosa cell adhesion during follicle assembly. The significant decrease in CDH3 expression at 90-dph, coupled with the presence of positive signals in the thecal epithelia of the medullary cavities (Fig. 4C, right panel), suggests its involvement in the disruption of medullary cavities and infiltration of the cortex, potentially affecting the activation of primordial follicles into primary follicles via the modulation of medullary–cortical spatial dynamics.

### cDNA cloning and characterization analysis

**Figure 5:**
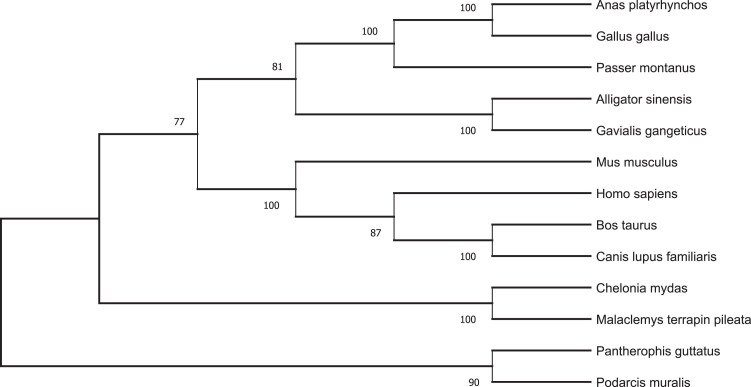
Neighbor-joining phylogenetic tree of CDH3 amino acid sequences.

A complete ORF of 2,352 bp encoding a precursor protein of 783 deduced amino acids from the CDH3 coding sequence (CDS) sequence was cloned. The predicted amino acid sequence contains three putative Ca^2+^-binding sites (cadherin repeat-like domains), 2 cadherin domains, and a cadherin cytoplasmic region (Supplementary Figure S4 of the Supplementary Information). This finding suggests that CDH3 may have a similar function with regard to cell adhesion that has been reported for members of the mammalian CAM family of genes. The CDH3 amino acid sequences of different species are grouped together in phylogenetic trees on the basis of their evolutionary relationships (Fig. 5). *Alligator sinensis* and *Crocodylus siamensis*, which are both members of the Crocodilian class, form a branch that clusters with avians. These findings indicate that reptilian CDH3 may be more closely related to that of birds than to that of other mammals and amphibians.

### CDH3 regulates oogenesis in Chinese alligator

**Figure 6:**
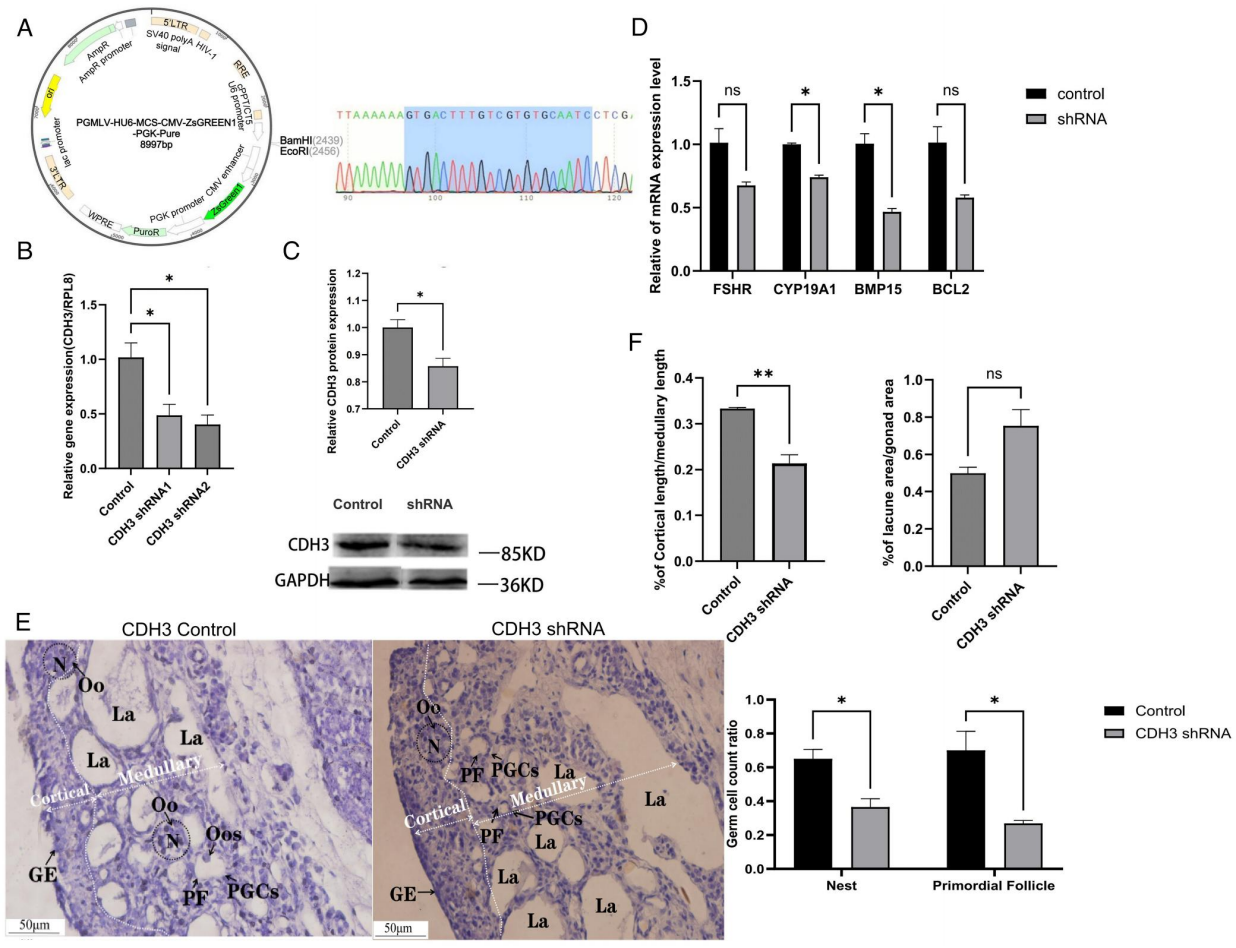
CDH3 regulate oogenesis in the Chinese alligator: (A) Constructions of RNA interference vectors. (B) Inhibition efficiency detection of CDH3 by RT-qPCR. shRNA(1:1E7TU,2:1E8TU). (C) Inhibition efficiency detection of CDH3 by Western blot. (D) mRNA expression of FSHR, CYP19A1, BMP15, and BCL2 in gonadal tissue transfected with shRNA2. (E) Histological sections of gonadal tissues following CDH3 Inhibition. N, Nest; Oo, Oogonia; La, Lacune; Oos, Oocytes; GE, germinal epithelium; PF, primordial follicle; PGCs, pre-granulosa cells. (F) Analysis of CDH3 gene expression impact on ovarian tissue architecture and germ cell quantification. *P < 0.05; **P < 0.01, ***P < 0.001.

The gonadal tissues were transfected with vectors harbor sequences designed for the interference of CDH3 (Fig. 6A). ShRNA transfection significantly reduced the mRNA expression and protein levels of CDH3 (*P* < 0.05; Fig. 6B,C). This decline was accompanied by simultaneous reduce in the mRNA expression levels of genes intricately associated with oogenesis (FSHR, CYP19A1, BMP15, and BCL2), as depicted in Fig. 6D (*P* < 0.05). As shown in Fig. 6E,F, the results showed that compared with the control group, after knockdown, the ratio of cortex to medulla increased, and the proportion of germ cells in the nest decreased, while the proportion of primordial follicles in the nest decreased (*P* < 0.05; Fig. 6F). These observations indicate that the suppression of CDH3 expression had a disintegrative effect on nests, as manifested by the increased proportion of germ cells residing within primordial follicles. This was accompanied by an expansion in both the relative area of the medullary compartment and the fractional cavity space, thereby fostering enhanced spatial adjacency between primordial follicles and the medullary zone. We postulate that this improved spatial organization facilitates the augmentation of the initial phases of primordial follicle maturation.

### Screening of miRNAs targeting cadherin family genes

**Figure 7:**
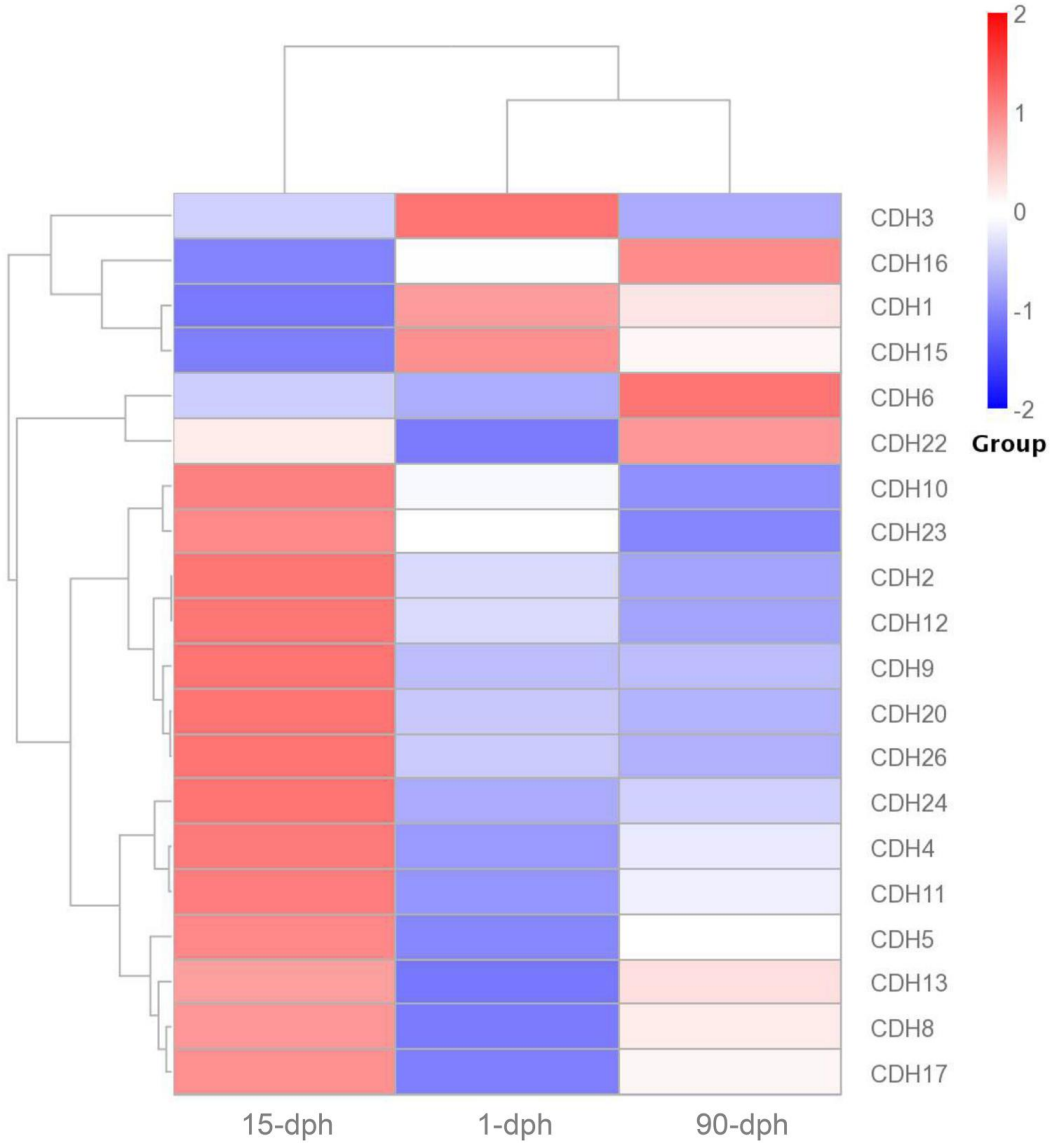
The hierarchical clustering digram of the differential miRNA expression level: right: different miRNA name.

In the cluster analysis library, miRNAs with similar expression patterns within the cell adhesion family will cluster together (Fig. 7), with the names of the corresponding cell adhesion family genes listed on the right side. Most of these miRNAs are highly expressed during the 15-dph period, while their transcription levels are lower during the 1-dph and 90-dph periods.

**Figure 8:**
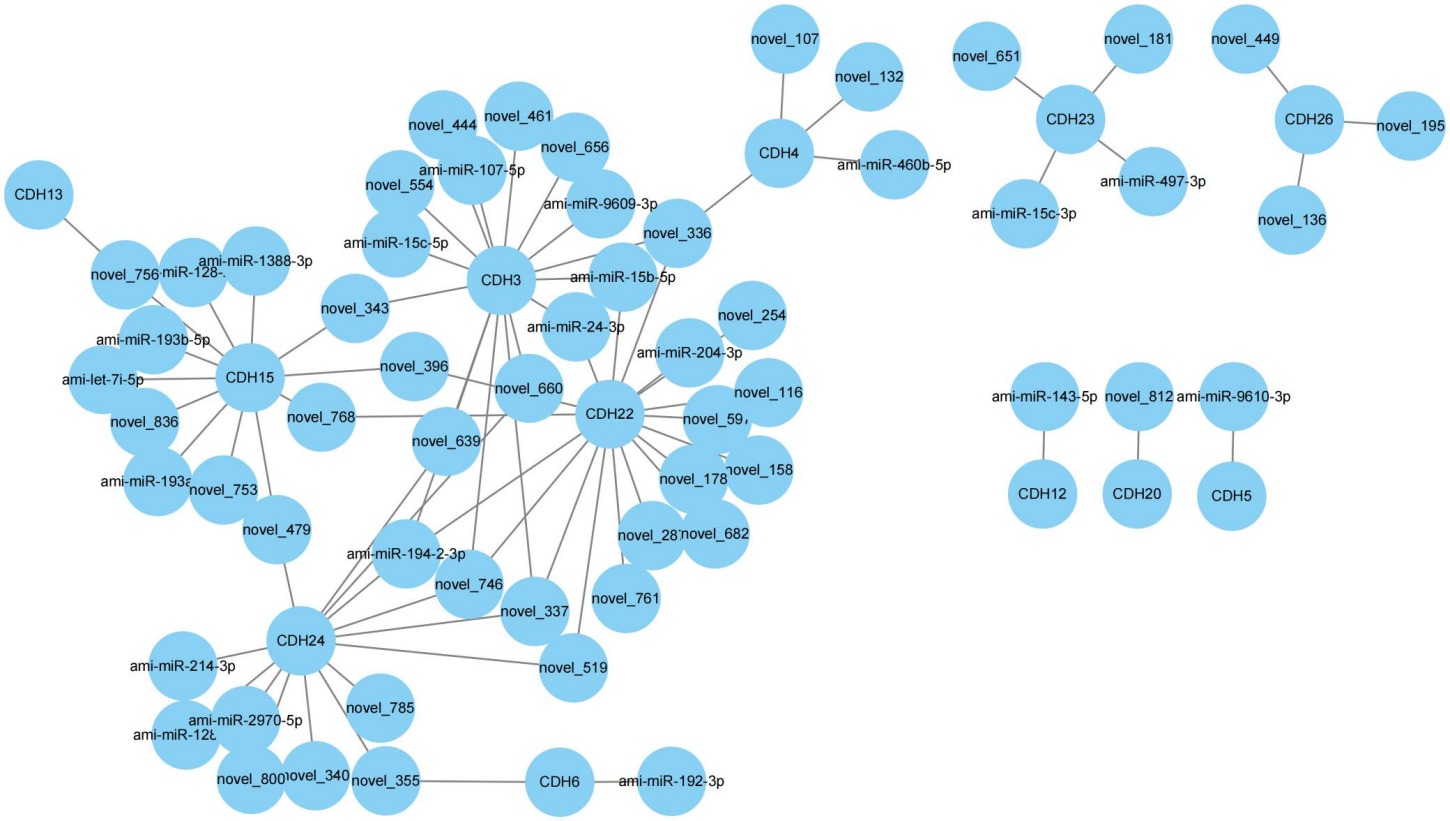
miRNA prediction of mRNA targeting relationships of Cadherin family genes.

As illustrated in Fig. 8, miRNAs targeting cadherin family genes were predicted with miRanda and RNAhybrid software. Our findings indicate that concerted regulation of multiple cadherin family genes, including CDH3, CDH4, CDH15, CDH22, CDH23, CDH24, and CDH26, is mediated by a consortium of miRNAs. Specifically, miR-194-2-3p, novel-343, novel-479, and novel-519 can concomitantly modulate the expression of multiple cadherin genes. Additionally, miR-9610-3p specifically targets CDH5, miR-143-5p affects CDH12, and novel-812 targets CDH20. Moreover, both miR-24-3p and miR-9609-3p demonstrated targeting affinity for CDH3. These findings provide insights for future research.

## Discussion

Oogenesis is an important reproductive process in animals, which enables offspring production through female gamete production. As an important post-transcriptional regulatory mechanism for gonadal development, miRNAs have been reported in the gonads of several reptiles (Lin et al. 2020b; Ma et al. 2020, 2021; Hu et al. 2022; Gao et al. 2024). Here, we performed sRNA sequencing to analyze miRNA expression in the gonads of Chinese alligators at 1 day, 15 days, and 90 days post-hatching, and constructed miRNA expression profiles accordingly.

As shown by the heat map, we observed that ami-miR-101-3p had the highest abundance only at 1-dph. Research shows that the high expression of miR-101-3p in goats results in the inhibition of granulosa cell proliferation (An et al. 2020). miR-101 regulates oocyte maturation in vitro by targeting HAS2 in porcine cumulus cells (Luo et al. 2022) since granulosa cells are an important part of the oocyte developmental process. miR-101-3p may regulate oogenesis in female Chinese alligators during the early stages by influencing the proliferation of granulosa cells. Moreover, we observed a decreasing trend in the level of miR-133b-3p across the 3 stages. miR-133b-3p can impact estrogen synthesis by targeting foxl2 in mice (Dai et al. 2013), and it is involved in early oogenesis in tilapia via the modulation of tagln2 (Ma et al. 2021). Therefore, miR-133b-3p may be involved in oogenesis via its regulation of the expression of sex hormone genes. Additionally, miR-10a-5p levels increase sequentially in 3 periods. In chickens, miR-10a-5p regulates granulosa cell proliferation and progesterone synthesis (Li et al. 2022). Meanwhile, miR-10a-5p has been shown to impact ovarian cancer cell survival and migration (Liu et al. 2021a). The process of oocyte maturation is inhibited by miR-10a-5p in human follicular fluid (Zhang et al. 2021b). These findings suggest that miR-10a-5p has important functions in the development of oogenesis in the Chinese alligators and may play a regulatory role in the progression rate of oogenesis.

CAMs likely play crucial roles in oocyte development, specifically in maintaining oocytes within germ cell cysts (Pepling 2012). Recent work utilizing the hamster model has implicated N- and E-cadherin in follicle formation, suggesting that CAMs may play a crucial role in adhesion between oocytes and granulosa cells during primordial follicle formation (Wang and Roy 2010). We observed not only that the expression levels of CDH3 were significantly higher than those of the other family members but also that CDH3 had a high expression phase during the embryonic stage, which is consistent with the biological phenomenon of mammalian oogenesis starting at this stage (Tingen et al. 2009).

CDH3 (P-cadherin) was described for the first time in 1986 (Nose and Takeichi 1986). CDH3 regulates cellular homeostatic processes that participate in embryonic development and maintain adult tissue architecture, playing a crucial role in cell differentiation, cell shape, cell polarity, growth, and migration (Larue et al. 1996; Raymond et al. 2009; Cavallaro and Dejana 2011). CDH3 expression increased but then decreased at 1-dph, 15-dph, and 90-dph. Drawing from these observations, we postulate that, when expressed at low levels, CDH3 potentially functions in preserving the structural integrity of nests at 1-dph. When CDH3 is highly expressed, however, its role appears to shift toward supporting the structural integrity of the newly established primordial follicles at 15-dph. The diminished expression of CDH3 in primordial follicles at 90-dph might be associated with the transformation from primordial to primary follicles. In this study, we observed that the knockdown of CDH3 affected the morphology of gonadal tissues and genes related to oogenesis. These findings suggest that CDH3 may play a role via a specific mechanism in regulating the oogenesis process. Previous studies have shown that the process of oogenesis is regulated by follicle-stimulating hormone receptor (FSHR) (Zhang et al. 2015), cytochrome P450 family 19 subfamily A member 1 (CYP19A1) (Blázquez et al. 2008; Esterhuyse et al. 2008), bone morphogenetic protein 15 (BMP15) (Di Pasquale et al. 2006; Layman 2006; Dranow et al. 2016), and B-cell lymphoma 2 (BCL2) (Aitken et al. 2011). Mechanistically, the overexpression of CDH3 resulted in increased expression levels of oogenesis-related genes (FSHR, CYP19A1, BMP15, and BCL2).

Moreover, the interference of CDH3 resulted in a decreasing trend in both the number of nests and the number of primordial follicles. These findings suggest that the downregulation of CDH3 leads to the disintegration of nests. Furthermore, the reduced CDH3 expression levels may hinder the effective formation of primordial follicles. The inability of oocytes released from nests to be enclosed by precursor granulosa cells may predispose them to apoptosis. These results indicate that CDH3 plays an important role in the stability of nests and the formation of primordial follicles in Chinese alligators.

In conclusion, we identified miRNAs in the gonads of female Chinese alligators; analyzed their differential expression at 1-dph, 15-dph, and 90-dph and further investigated the role of miRNAs in the oogenesis process of the Chinese alligator. By screening the target genes of the differentially expressed miRNAs, we showed that CDH3 plays an important role in maintaining the stability of nests and promoting the formation of primordial follicles. These results will further inform our understanding of the mechanisms involved in oogenesis. Furthermore, that this research is expected to contribute to the conservation of the Chinese alligator.

## Supplementary Material

zoaf058_Supplementary_Data
