# Supplementary material for: MicroRNA expression profiling and functional analysis of CDH3 during oogenesis in the Chinese alligator (Alligator sinensis)
Source: Curr Zool. 2025 Aug 21;72(3):428–40. doi: 10.1093/cz/zoaf058 (PMC13290405; doi:10.1093/cz/zoaf058)
Supplement: zoaf058_Supplementary_Data [file zoaf058_supplementary_data.docx]

**Table S1 Primers used for cloning cDNA of the Chinese alligator CDH3.**

| **Gene** | **Primer (5'-3')** | **Product (bp)** |
| --- | --- | --- |
| CDH3F1 | CATTCGAGCCCACAGACACC | 233 |
| CDH3R1 | TCTCAGGGACTCGGATTGG |  |
| CDH3F2 | TCGTCCCTCCAATCCGAGTC | 399 |
| CDH3R2 | CTGCATCCGTGGCGTTCA |  |
| CDH3F3 | GGGAGAACATTGACAAATACCA | 487 |
| CDH3R3 | CACCTCTGCCGCATACATC |  |
| CDH3F4 | AGATGTATGCGGCAGAGGTG | 396 |
| CDH3R4 | GCGTAGGTGGCGATGGTCT |  |
| CDH3F5 | ACCATCGCCACCTACGC | 698 |
| CDH3R5 | CTCCTTCCCTGACTTCCTCC |  |
| CDH3F6 | AGCCGCGTGACTTTGTCG | 1212 |
| CDH3R6 | GGATGGTGCAGGGAATGGT |  |

**Table S2 Details of primers information used for RT-PCR**

|  | **Primer sequences (5'-3')** | | | **Production size (bp)** |
| --- | --- | --- | --- | --- |
| **Genes** | **Accession number** | **Forward** | **Reverse** |  |
| CDH1 | XM_025209137. | GCCGTGATTGAGGTCGTTGA | GCGGTGGAAGTCGGGAGAT | 335 |
| CDH2 | XM 025214267 | GAAGCCCATAGACTTTGAGA | GGGTCCTGAGCCGTGAA | 231 |
| CDH3 | XM 025209139 | TCGTCCCTCCAATCCGAGTC | CTGCATCCGTGGCGTTCA | 399 |
| CDH4 | XM_00602592 | GGAACGGCATACGAAGC | TGGAGGAGTTGAGGGAGC | 377 |
| CDH5 | XM_006016415 | TTTGTCCGTGGGTACTTC | GGAGGGCTTGATATTCG | 365 |
| CDH8 | XM 02521437 | CTCAGGCAGTTGATAGGG | TTCTTCTTTGGCTTCTCG | 311 |
| CDH10 | XM 025214327 | GCAGCACCAATACCCTA | TGTCCTCTTCTCCACCC | 206 |
| CDH12 | XM_006037154 | GGCTCCGAACCTCAGTA | CCAGAAACTTTGGCTCATT | 270 |
| Rpl8 | XM_006015645 | GGTGTGGCTATGAATCCTGT | ACGACGAGCAGCAATAAGAC | 280 |

**Table S3 Statistical results of the corresponding raw sequencing data quality for each sample.**

| Library | Sample | Reads | Bases | Error rate | Q20 | Q30 | GC content |
| --- | --- | --- | --- | --- | --- | --- | --- |
| FRRN210015686-1a | 1-dph | 13325519 | 0.666G | 0.01% | 99.52% | 98.00% | 48.51% |
| FRRN202260881-1a | 15-dph | 13415115 | 0.671G | 0.01% | 99.39% | 97.80% | 48.51% |
| FRRN210210833-1a | 90-dph | 20892958 | 1.045G | 0.01% | 99.38% | 97.57% | 51.72% |

Reads: the number of sequencing sequences for each raw sequencing file; Bases: The number of sequencing sequences multiplied by the length of the sequence and converted to reads in G; Error rate: sequencing error rate; Q20: percentage of bases greater than 20; Q30: bases greater than 30 for total bases; GC content: number of bases G and C and percentage of total bases.

**Table S4 Cross-species analysis results of miRNA families**

| Species | let-7 | | miR-10 | miR-101 | | miR-125 | miR-133 | | miR-143 | | miR-145 | miR-155 | | miR-16 | | | miR-182 | miR-184 | miR-203 | | miR-30 | | miR-383 | | | miR-9 | miR-92 | | | miR-9609 | | miR-99 |
| --- | --- | --- | --- | --- | --- | --- | --- | --- | --- | --- | --- | --- | --- | --- | --- | --- | --- | --- | --- | --- | --- | --- | --- | --- | --- | --- | --- | --- | --- | --- | --- | --- |
| Alligator mississippiensis | + | | - | - | | - | - | | + | | + | + | | - | | | + | + | + | | - | | + | | | - | - | | | + | | - |
| Anolis carolinensis | + | | - | - | | - | - | | + | | + | + | | - | | | + | + | + | | - | | + | | | - | - | | | - | | - |
| Artibeus jamaicensis | + | | - | - | | - | - | | + | | + | - | | - | | | - | - | - | | - | | - | | | - | - | | | - | | - |
| Bos taurus | + | | - | - | | - | - | | + | | + | + | | - | | | + | + | - | | - | | + | | | - | - | | | - | | - |
| Canis familiaris | + | | + | - | | - | - | | + | | + | + | | - | | | + | + | + | | - | | + | | | - | - | | | - | | - |
| Capra hircus | + | | - | + | | - | - | | + | | + | + | | - | | | + | + | - | | - | | + | | | + | - | | | - | | - |
| Cavia porcellus | + | | - | - | | - | - | | + | | + | + | | - | | | + | + | + | | - | | + | | | - | - | | | - | | - |
| Chrysemys picta | + | | - | - | | - | - | | + | | + | - | | - | | | + | + | + | | - | | + | | | - | - | | | - | | - |
| Columba livia | + | | - | - | | - | - | | + | | + | + | | - | | | + | + | - | | - | | + | | | - | - | | | - | | + |
| Cricetulus griseus | | + | - | | - | - | | - | | + | - | | + | | + | + | | + | | - | | - | | - | + | | | - | - | | - | |
| Danio rerio | | + | - | | - | - | | - | | + | + | | + | | - | + | | + | | - | | - | | - | - | | | - | - | | - | |
| Drosophila ananassae | | + | + | | - | + | | + | | - | - | | - | | - | - | | + | | - | | - | | - | - | | | - | - | | - | |
| Gallus gallus | | + | - | | + | - | | - | | + | + | | + | | - | + | | + | | + | | - | | + | - | | | + | - | | - | |
| Gorilla gorilla | | + | - | | + | - | | - | | + | + | | + | | + | + | | - | | - | | - | | + | + | | | - | - | | - | |
| Ophiophagus hannah | | + | - | | - | - | | - | | + | + | | - | | - | + | | - | | + | | - | | + | - | | | - | - | | - | |
| Ornithorhynchus anatinus | | + | - | | - | + | | - | | + | + | | + | | - | + | | + | | - | | - | | + | - | | | - | - | | + | |
| Ovis aries | | + | - | | - | - | | + | | + | - | | - | | - | - | | - | | - | | - | | - | - | | | - | - | | - | |
| Python bivittatus | | + | - | | - | - | | - | | + | + | | - | | - | + | | + | | + | | - | | + | - | | | - | - | | - | |
| Taeniopygia guttata | | + | - | | - | - | | - | | - | - | | + | | - | - | | + | | + | | - | | + | - | | | - | - | | - | |
| Tupaia chinensis | | + | - | | + | - | | - | | + | - | | + | | - | - | | + | | - | | - | | - | - | | | - | - | | - | |
| Xenopus laevis | | + | - | | + | - | | - | | + | + | | - | | - | - | | + | | + | | - | | - | - | | | - | - | | + | |
| Xenopus tropicalis | | + | - | | - | - | | - | | + | + | | + | | - | + | | + | | + | | - | | + | - | | | - | - | | + | |


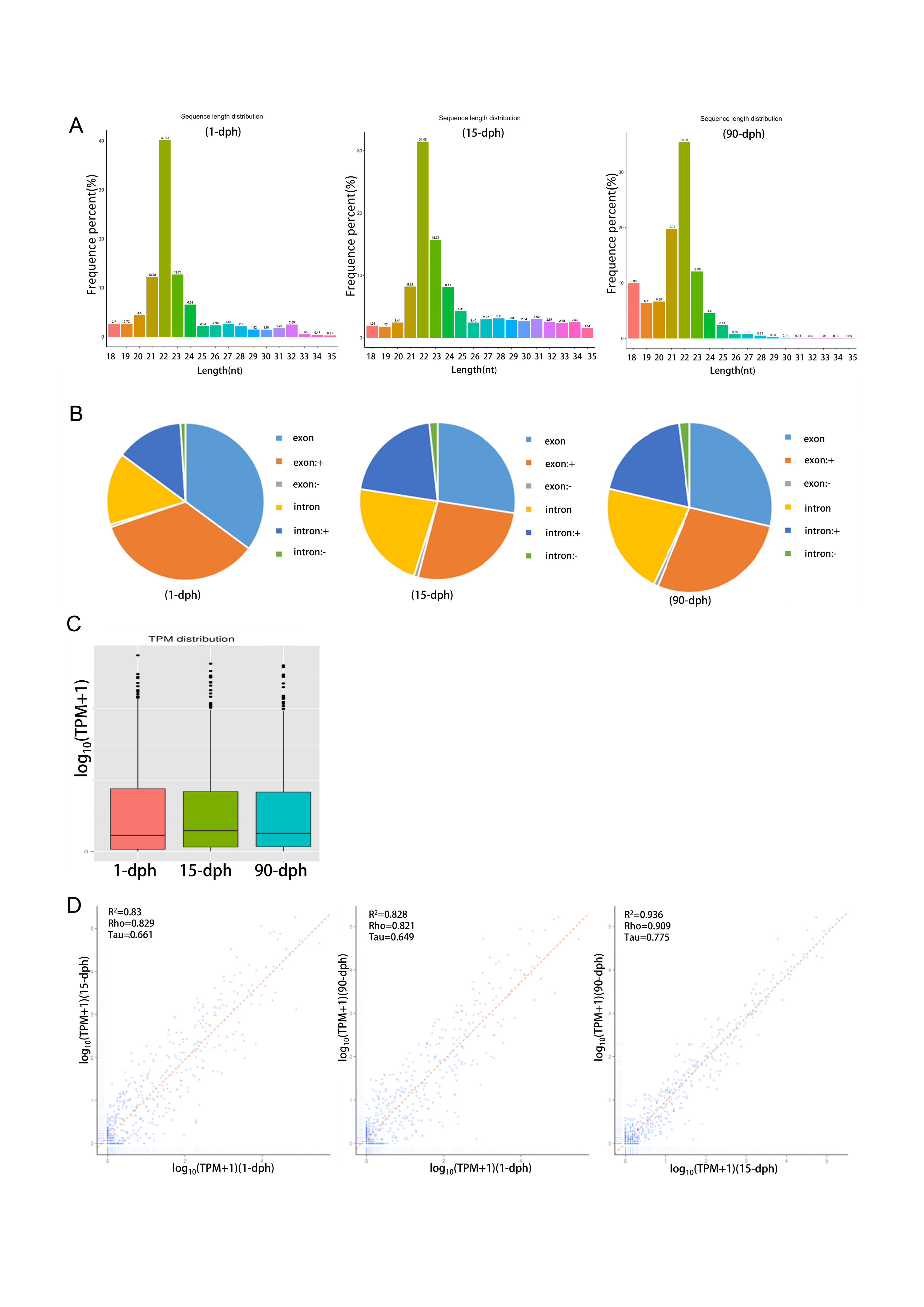


**Figure S 1. Analysis of miRNA differential expression during the 1-dph, 15-dph, and 90-dph periods:** (A) Statistical analysis of sRNA species, abundance, and length distribution within adefined length range. (B) sRNA mapping onto gene exons and introns across different samples. (C) The TPM values of miRNAs at 1-dph,15-dph, and 90-dph. (D) miRNA expression correlation between 1-dph vs 15-dph,1-dph vs 90-dph, and 15-dph vs 90-dph.


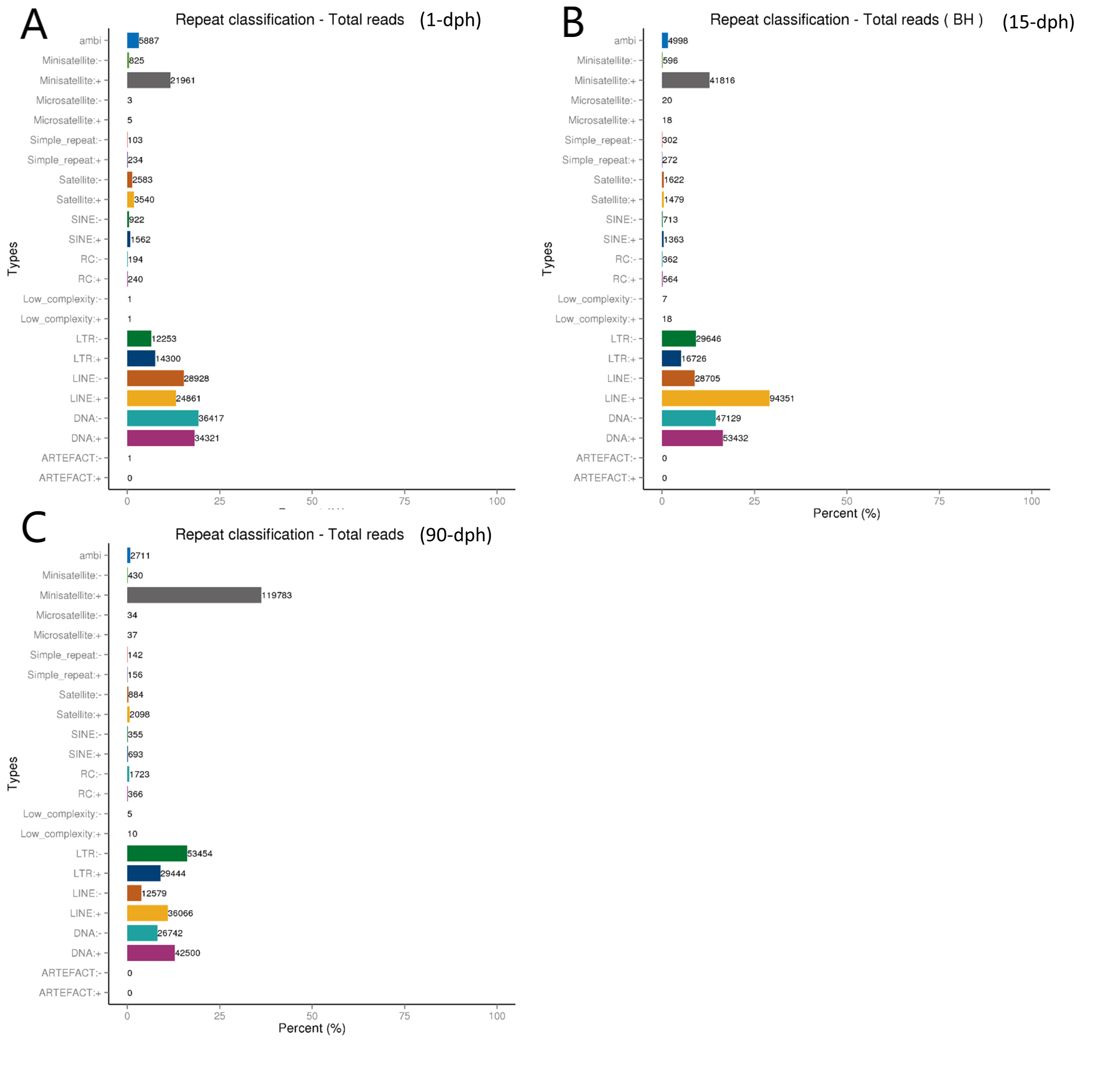


**Figure S 2 Small RNA classification annotation based on repetitive sequence information** (A) Classification and annotation of small RNA (1-dph). (B) Classification and annotation of small RNA (15-dph). (C) Classification and annotation of small RNA (90-dph).

**
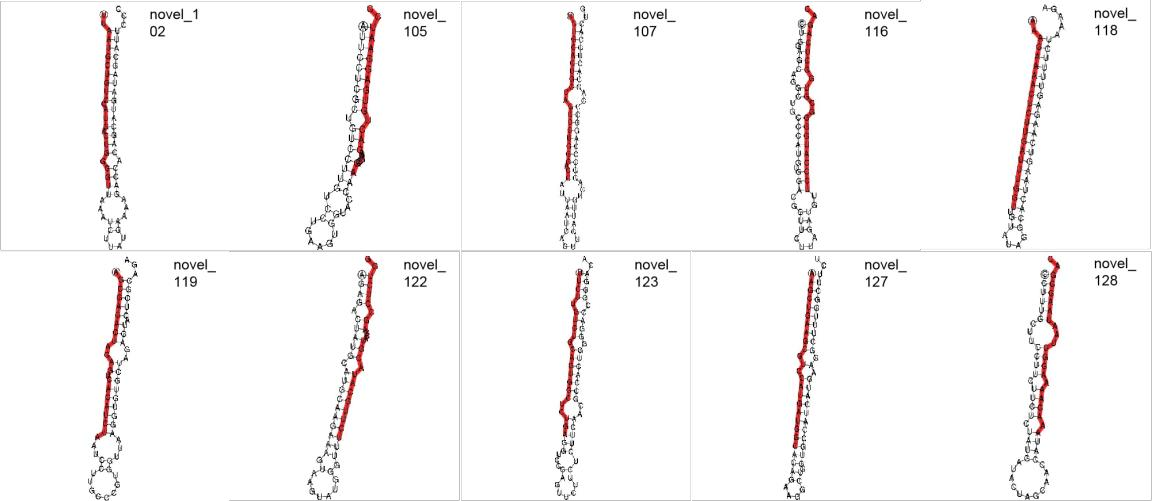
**

**Figure S 3** **New miRNA secondary structure**

The predicted secondary structure of novel miRNA


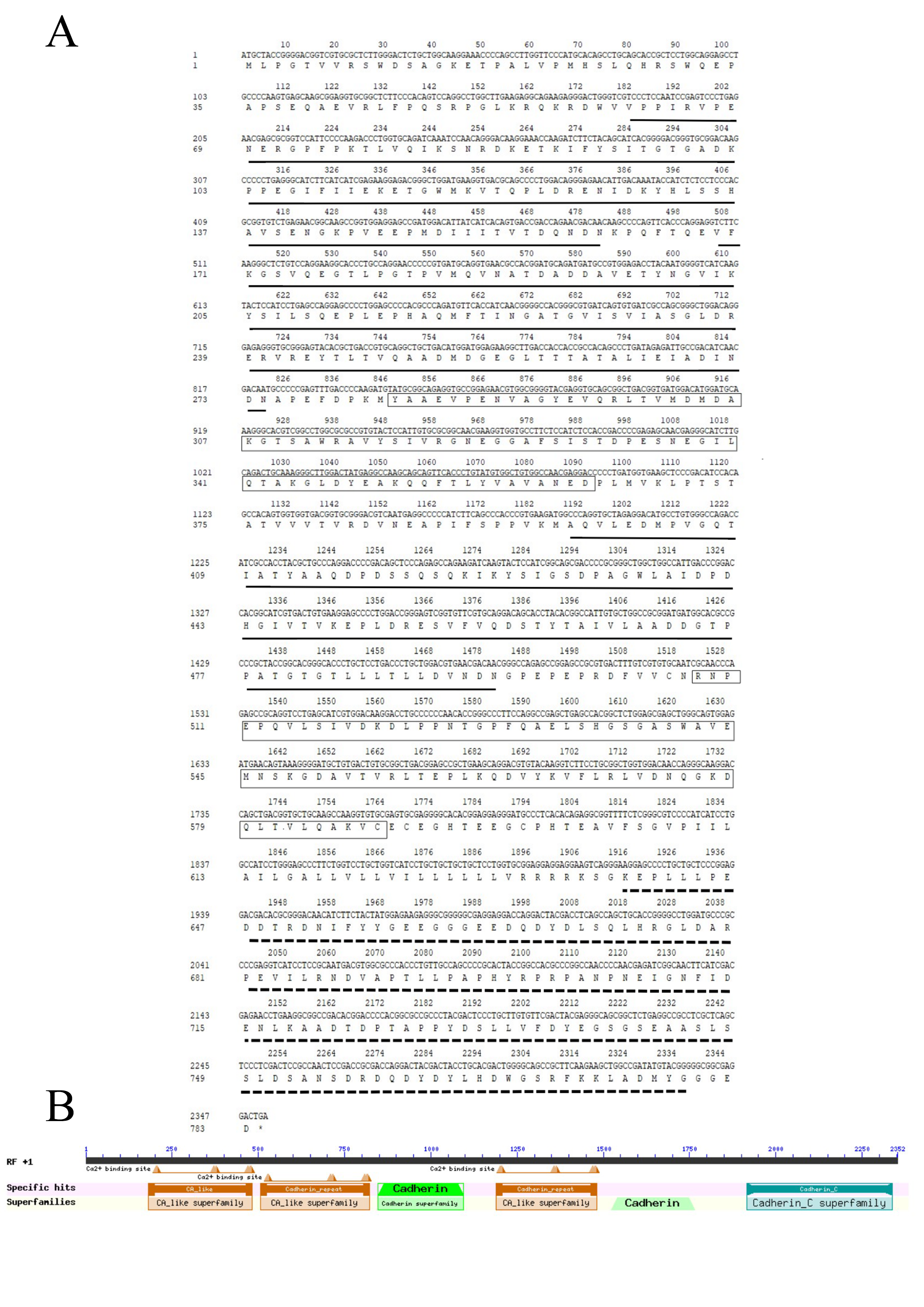


**Figure S 4 . Structure and sequence analysis of CDH3.** (A)The nucleotide sequence and the deduced amino acid sequence of CDH3. (B) CDH3 conserved domain prediction
